# Functionalized Nanostructures with Application in Regenerative Medicine

**DOI:** 10.3390/ijms13033847

**Published:** 2012-03-22

**Authors:** Macarena Perán, María A. García, Elena López-Ruiz, Milán Bustamante, Gema Jiménez, Roberto Madeddu, Juan A. Marchal

**Affiliations:** 1 Department of Health Sciences, University of Jaén, Jaén E-23071, Spain; E-Mails: mperan@ujaen.es (M.P.); elruiz@ujaen.es (E.L.-R.); 2 Research Unit, Hospital Universitario Virgen de las Nieves, Granada E-18014, Spain; E-Mail: mangelgarcia@ugr.es; 3 Biosciences Institute, University College Cork, Cork, Ireland; E-Mail: 111224080@umail.ucc.ie; 4 Biopathology and Regenerative Medicine Institute (IBIMER), Biomedical Research Centre, University of Granada, Granada E-18100, Spain; E-Mail: gemajg@correo.ugr.es; 5 Department of Biomedical Sciences, University of Sassari, 07100 Sassari, Italy; E-Mail: rmadeddu@uniss.it; 6 Department of Human Anatomy and Embryology, Faculty of Medicine, University of Granada, Granada E-18012, Spain

**Keywords:** regenerative medicine, nanomaterials, scaffolds, cell therapy, stem cells, bone, cartilage, cell encapsulation, cell tracking, gene and drug delivery

## Abstract

In the last decade, both regenerative medicine and nanotechnology have been broadly developed leading important advances in biomedical research as well as in clinical practice. The manipulation on the molecular level and the use of several functionalized nanoscaled materials has application in various fields of regenerative medicine including tissue engineering, cell therapy, diagnosis and drug and gene delivery. The themes covered in this review include nanoparticle systems for tracking transplanted stem cells, self-assembling peptides, nanoparticles for gene delivery into stem cells and biomimetic scaffolds useful for 2D and 3D tissue cell cultures, transplantation and clinical application.

## 1. Introduction

Regenerative medicine is a broad interdisciplinary science that attempts to restore lost, damaged, or aging cells and tissues to a state as close as possible to its native architecture and function. Therapeutic approaches in regenerative medicine may be divided into: (i) cell-based; (ii) biomaterials-based or (iii) combined tissue engineering strategies. Over the past twenty years, there have been tremendous advances in the fields of stem cell biology, nanotechnology and bioengineering, the intersection of which offers increasing potential to achieve the goal of truly regenerative therapies for myriad pathologies.

Stem cells are defined by their ability to retain their stem cell capacity through controlled proliferation (self-renewal) while also dividing to produce differentiated daughter cells, which lose some degree of “stemness” and proceed to form mature cells of all lineages in the body [1]. These important functional properties of SCs, which are critical to maintaining organ homeostasis, underlie the tremendous therapeutic potential for SCs to effect tissue and organ regeneration. Stem cells are classified based on their source tissue and their potency, which refers to the potential of a given stem cell to differentiate into mature progeny by the goal of reconstructing, repairing, or replacing missing or damaged tissue [2].

Nanotechnology is defined by the size of a material (generally 1–100 nm) or manipulation on the molecular level, and it involves a broad range of nanoscaled materials used in various fields of regenerative medicine, including tissue engineering, cell therapy, diagnosis and drug and gene delivery. Moreover, the growing interest and expansion in nanomedicine during the last years has led to new perspectives and advances in biomedical research as well as in clinical practice. To date, nearly 30 nanotechnology-based products have been approved for clinical use, focused mainly on liposomal formulations and stealth polymer-drug conjugates. The application of nanotechnology tools to the development of structures at the molecular level enables the improvement of the interactions between material surfaces and biological entities. Thus, nanomedicine provide the possibility to produce surfaces, structures and materials that can mimic the natural environment of cells, to promote certain functions, such as cell adhesion, cell mobility and cell differentiation [3].

Since Langer and Vacanti in 1993 [4] proposed the combined use of stem cells, scaffolds, and inductive factors as the basis for tissue engineering, researchers have been able to fabricate increasingly complex tissue/organ constructs and some are used clinically today as standard treatment for a variety of conditions. Scaffolds are processed in order to produce 3D structures, with proper shape, size, architecture, and physical properties, tailored to fulfil specific functions. Therefore, tissue engineering products are designed to mimic tissue architecture and responses. So, key scaffold requirements are biocompatibility, controlled porosity and permeability, suitable mechanical and degradation kinetics properties comparable to the targeted tissue and, additionally, support for cell attachment and proliferation by the addition of nanotopographies to the biomaterial surface [5,6].

Natural or synthetic materials are used to make scaffolds and depending on the final purpose, barriers (membrane or tubes), gels or 3D matrices are developed to mimic the extracellular environment of a target tissue or organ. Natural materials are derived from human or animal (xenogeneic) sources and are composed of extracellular components [7]. They include collagen, silk protein, Matrigel, small intestinal submucosa, agarose, alginate and chitosan [8–11]. Examples of natural scaffolds that have been applied clinically include decellularized dermis to treat burn injuries [12] and decellularized small intestine ureter, or xenogeneic vessels to restore vascular function [13]. Although these materials have shown promising results in tissue repair, they have some drawbacks regarding mechanical properties, degradation, immunogenicity and cross-contamination. Synthetic scaffolds have been constructed using synthetic materials or a combination between natural and synthetic materials. Polyhydroxic acids, polytetrafluoroethylene, steel titanium, or ceramics are examples of synthetic polymers with improved biocompatibility [14,15]. Natural materials, such as collagen, gelatine, chitosan, alginates and silk or synthetic poly(lactic acid) (PLA), poly(lactic-co-glycolic acid) (PLGA), poly-epsiloncaprolactone (PCL), or polyvinyl alcohol (PVA) polymers, are the most common materials employed for the fabrication of nanofibre scaffolds. These matrices can be created with high structural precision, using complex polymers and assembly techniques, to control material properties such as stiffness, degradation and porosity. The advent of nanotechnology has allowed further developments in the field of biomaterials. Suitable nano-modified surfaces create a nanotopography which facilitates cell adhesion and can induce a better cellular response and specific cell differentiation than untreated surfaces [16]. For example, nano-structured PLGA surfaces have been shown to accelerate chondrocyte adhesion and proliferation, as well as extracellular matrix production [17,18]. Furthermore, vascular graft (PLGA) and stent (titanium) surfaces with nanometer surface roughness improve endothelial cell functions as compared to nano-smooth polymer and titanium surfaces [19,20].

Another promising strategy for tissue regeneration is the use of nanomaterials as cell delivery vehicles. The most commonly used nanomaterials are peptide amphiphiles, self-assembled peptides, carbon nanotubes (CNTs), electrospun fibers and layer-by-layer structures [21]. The development of novel nanostructures formed by bioactive molecules that can interact specifically and reproducibly with cell receptors and proteins to control processes such as cell survival, cell proliferation, cell differentiation, and dedifferentiation in the context of tissue and organ regeneration has generated enormous interest. Many research groups are currently taking advantage of the better understanding of molecular self-assembly and nanoscience to develop bioactive, biomimetic, and multifunctional materials for regenerative medicine [22–25]. One advantage of functionalizing nanomaterials over materials in general is the fact that the functionalization could be performed at the level of the nanostructural unit, to be amplified in a predictable manner as the material is built up via a bottom-up approach. Later advances in this field is the generation of bioactive and biodegradable nanoscale filaments that mimic those in extracellular matrices and can display in tuneable densities peptide signals that promote regenerative processes [26–29].

Controlled release of biomolecules is crucial in the support and enhancement of tissue growth in tissue engineering applications. Nanotechnology approaches in delivery systems can enhance the success of specific therapeutic agents, such as growth factors, proteins, peptides, DNA, RNAi and small drugs, which are of vital importance for tissue regeneration [30].

Further progress in cell therapy leading to clinical trials requires the crucial use of non-invasive techniques for monitoring the efficacy of cell therapy and graft survival in the host organism. For pre-clinical and clinical trials, it will be important to track SCs noninvasively in order to evaluate their therapeutic effect and grafting location to rule out potentially dangerous side effects. Moreover, nanoparticle (NP)-labeled SCs/progenitor cells might contribute to our understanding of the cell migration processes in numerous diseases, such as neurologic diseases, myocardial infarction and cancer [31]. In addition, this platform might give us important information and cues about the differentiation program of SCs. NP systems for tracking transplanted SCs have been developed using a variety of non-invasive imaging techniques including optical imaging (luminescence, photoacoustic tomography and optical coherence tomography) [32,33], magnetic resonance imaging (MRI) [34,35] and radionuclide imaging (PET and SPECT) [36–38].

The aim of this review is to highlight some of the most recent advances in the exciting fields of nanotechnology and regenerative medicine. We will focus in the functionalization of nanostructured biomaterials with potential applications in regenerating different tissues. Therefore, the themes covered in this article include: (i) nano- or micro-fabricated scaffolds with different biomolecules (depending on the targeted cells) and nano-modified surfaces; (ii) stem cell encapsulation with nano-fabrication techniques; (iii) NP systems for tracking transplanted SCs using several imaging modalities; and (iv) NPs for gene and drug delivery targeting SCs. We also emphasize some of the ways they are being used in translational and clinical practice.

## 2. Nanostructure Scaffolds for Tissue Engineering

A main strategy of regenerative medicine is the construction of a biocompatible scaffold that, in combination with living cells and/or bioactive molecules, replaces, regenerates or repairs damaged cells or tissue. Numerous studies, using nanostructured materials, have demonstrated the validity of this approach for a variety of cell types and the application in regenerating different tissues (such as cardiac, bone, cartilage, skin, bladder, nervous and vascular) by enhancing the biological properties of the cells such as cell adhesion, cell mobility and cell differentiation [3,39–42]. In the *in vivo* condition the cells are located in three-dimensional (3D) microenvironments, where they are surrounded by other cells and by the extracellular matrix (ECM), whose components, such as collagen, elastin, and laminin, are organized in nanostructures (*i.e.*, fibers, triple helixes, *etc.*) with specific bioactive motifs that regulate the cell homeostasis. It is therefore essential to develop scaffolds that create synthetic microenvironments providing 3D support, so as to control and direct the cellular behavior and to promote specific cell interactions [43,44]. The goal of nanomaterial-directed stem cell culture is to mimic the properties, both physical and biochemical, of the physiological stem cell niche. Electrospinning is a widely used technique for the production of nanofibers that offers great flexibility in terms of the choice of scaffold material, as well as finer control over the scaffold geometry [45–47]. In the electrospinning process, nanofibres are created as polymeric jets from the surface of a polymeric solution in a high intensity electrostatic field when the electric field overcomes the polymer surface tension. Modulation of spinning parameters such as flow rate and collecting distance, and polymer solution properties such as solvent, concentration, conductivity, and surface tension, properties of the resultant nanofibrous meshes, such as fibre diameter, porosity, mechanical properties and surface topography can be easily controlled [48,49]. Moreover, additional functionalities can be incorporated via protein coatings, or chemical conjugation of specific signalling molecules with great utility in SCs-based therapy [50]. The development of these nanoscaffold-based therapy methods in combination with SCs is an important tool for tissue engineering and regenerative medicine.

### 2.1. Nanoscaffolds Used in Regeneration of Hard Tissues

The ideal situation for hard tissue regeneration is to utilize “bioactive” materials that stimulate active tissue regeneration. Substrate rigidity or flexibility has been shown to regulate cell behaviour and is a critical determinant of tissue function [51]. Stiffer scaffolds are generally more suitable for load-bearing bone or cartilage engineering.

#### 2.1.1. Bone Regeneration

The bone tissue is a mineralized organic matrix mostly formed from collagenous fibres and calcium phosphate in the form of hydroxyapatite (HA), with embedded osteoblasts, osteocytes and osteoclasts as cell components. For bone reconstruction, nanoscaffolds have been provided with suitable biophysical properties, such as hardness and porosity, as well as support for cell growth and differentiation. In the past several years, various nanofibrous matrices have been created. Most of the nanoscaffolds produced emulate the structural, compositional and biological characteristics of natural bone. They are based primarily on nano-HA, collagen, electro-spun silk, anodized titanium and nano-structured titanium surfaces [52–55]. Nanofibres have been shown to promote osteogenesis and biomineralization with primary osteoblasts [56–58]; however, the use of primary osteoblasts is limited by (i) the restricted availability and inherent donor site morbidity; (ii) their limited proliferative capacity; (iii) the age dependent behavior; or (iv) the risk of dedifferentiation during *in vitro* culture.

On the other hand, SCs are an attractive option because their large proliferative capacity and their ability to differentiate into multiple cell types [59]. Numerous studies have illustrated the ability of mesenchymal stem cells (MSCs) collected from several sources to differentiate into osteoblasts under osteogenic conditions on nanofibres. For instance, bone marrow-derived MSCs posses the ability to differentiate into bone tissue after seeding on natural (collagen or silk fibroin), degradable synthetic polymers (PLA, PCL and PLGA) as well as on a blend of synthetic and natural polymers such as gelatine, collagen, silk fibroin and chitosan [60–62]. In another study, a combination of bone morphogenic protein 2 (BMP-2) and HA NPs encapsulated into silk electrospun matrices was used to synergistically enhance bone formation from seeded bone marrow-derived human MSCs (hMSCs) [63]. Recently it has been shown the effects of functionalized nano-HA, PLGA, or nano-HA-PLGA composites, and a bone morphogenetic protein (BMP-7)-derived short peptide (DIF-7c) on osteogenic differentiation of bone marrow-derived hMSCs. Results showed that nano-HA and nano-HA-PLGA composites promoted an osteogenic differentiation of hMSCs, comparable with the differentiation obtained by direct injection of the DIF-7c peptide into the culture media. These findings could be eventually translated to clinical applications [64].

Other promising materials for bioengineering applications are carbon nanotubes (CNTs). CNTs are conductive and have nanostructured dimensions that mimic the 3D structure of proteins found in ECM. Large CNTs lead to a dramatic stem cell elongation, inducing cytoskeletal stress and selective differentiation into osteoblast-like cells [65]. Furthermore, hMSCs grown on CNTs networks could recognize the arrangement of individual CNTs in the CNT network. Namgung *et al.* showed that hMSCs on aligned CNTs networks exhibited enhanced proliferation and osteogenic differentiation compared to those on randomly oriented CNT networks due to mechanotransduction pathways triggered by high cytoskeletal tension in the aligned hMSCs [66]. In addition, surface engineering in carbon nanoscaffold such as carbon-coated TiO(2) nanotubes or functionalized PEG-conjugated multiwalled carbon nanotubes (MWCNT-PEG) sprayed onto plain coverslips induced higher levels of osteo-differentiation in hMSCs [67,68]. Engineering graphene consists in a two-dimensional structure comprising layers of carbon atoms arranged in six-membered rings [69], and might be a novel option for bone tissue regeneration.. Results have shown that proliferation and morphology of hMSCs were not affected after seeded on graphene films. Moreover in presence of an osteogenic medium, graphene coating helped by remarkably accelerating the differentiation of hMSCs at a rate comparable to differentiation under the influence of BMP-2 [70]. Lee *et al.* have suggested that the rapid osteogenic differentiation could be due to the ability of graphene to act as a platform for the accumulation and interactions of osteogenic inducers included in the conditioned medium such as dexamethasone and β-glycerolphosphate [71]. Specific interactions with other inducers (e.g., insulin) can block differentiation into other cell types. Moreover, some of these properties might be altered by varying the composition of graphene; for example, graphene oxide does not alter the structure of insulin and cells can differentiate into adipose tissue [71].

In two recent studies Seyedjafari *et al.* seeded HA and nano-HA coated and uncoated electrospun PLLA fibres with human cord blood derived SCs and implanted the scaffolds subcutaneously into mice [72,73]. After 10 weeks, scaffolds without HA showed no calcium deposition and were surrounded by a granulomatous inflammatory response while scaffolds with HA showed significant mineralization with little inflammatory response [72]. Additionally, higher order bone structures such as trabeculi and bone marrow were found within the newly formed ectopic bone [72,73].

Nanofibrous (NF) matrices have also been shown to enhance the expression of osteogenic genes and proteins and calcium staining of pluripotent embryonic stem cells (ESCs) [74]. Recently, Smith *et al.* examined the effects of the nanofibrous architecture in both two-dimensional (2-D) PLLA thin matrices and 3D PLA scaffolds to assess their effect on the osteogenic differentiation of human ESCs (hESCs) *in vitro* compared to more traditional solid films and solid-walled (SW) scaffolds. After 6 weeks of 3D culture, the hESCs on the NF scaffolds expressed significantly more osteocalcin mRNA compared to these on the SW scaffolds. The data indicates that the NF architecture enhances the osteogenic differentiation of the hESCs compared to more traditional scaffolding design [75]. In addition, homogenous hESCs-derived MSCs (hESCs-MSCs) have been generated and used to construct bone tissue. The hESCs-MSCs cultured on 3D NF PLLA scaffolds in combination with dexamethasone and BMP-7 stimulation *in vitro* showed highly mineralized tissues developed with specific bone marker genes expressed. These results indicate the promise of hESCs-MSCs for bone regeneration [76].

Reprogramming somatic cells into an ESC-like state, induced pluripotent stem (iPS) cells, has emerged as a promising new venue for customized cell therapies that avoided the controversy surrounding human ESCs. A recent study demonstrated the ability of murine iPS-derived osteoblasts seeded in a gelfoam matrix followed by subcutaneous implantation, in syngenic imprinting control region mice, to express matrices characteristic of bone [77]. Moreover, graphene and graphene oxide have the ability to act as platforms to support the iPSCs culture, exhibiting disparities in the differentiation propensity. Graphene hampered spontaneous differentiation towards the endodermal lineage whereas graphene oxide promoted differentiation along the endodermal pathway. These results underlined that the different surface properties of graphene and graphene oxide governed the iPSCs behavior, which have great potentials for regenerative medicine [78].

#### 2.1.2. Cartilage Regeneration

Cartilage is an avascular tissue composed of chondrocytes entrapped in an ECM rich in proteoglycans and collagens. Chondral defects suppose a challenging clinical problem as the proportion of elderly people in the population increase. Cartilage injuries lead to joint pain and loss of function with limited capacity for self-repair [79]. Innate repair mechanisms in cartilage are limited due to the scarcity/absence of resident SCs and the lack of a vascular and lymphatic system. Clinical treatments for articular cartilage injury include physical therapy, arthroscopic drilling, debridement, autologous osteochondral grafts from non-weight-bearing body regions, or autologous cell injections. However, the donor site morbidity and the difficulty in trimming and grafting for the desired shape limit their clinical applications [80].

Tissue engineering strategies have long been used for cartilage regeneration and have been based mostly on matrix seeded with either chondrocytes or MSCs [81]. Biomaterials, for instance, collagen, fibrin, alginate, chitosan, hyaluronic acid and polyesters have been employed into 3D exogenous ECMs for guiding cartilage regeneration [82]. NF scaffolds have introduced some advantages, such as high surface area, volume ratio and collagen fibre-mimetic nano-scale fibres, which can be translated into biologically favourable properties to enhance cartilage growth [83]. A recent study shows an increased chondrogenic differentiation and the production of ECM of BM-derived hMSCs seeded on electrospun PCL nanofiber meshes and cultured in a multichamber flow perfusion bioreactor [84].

The applicability of stem cell-seeded on NF scaffolds has also been evaluated *in vivo*. The capacity of cartilage regeneration of BM-derived hMSCs on PVA/PCL nanofibre scaffolds has been assayed after implantation into rabbit full-thickness cartilage. To improve the surface hydrophilicity, the biocompatible water-soluble synthetic biodegradable polymer PVA was selected to be electrospun concurrently with PCL via hybrid electrospinning. Results showed that PVA/PCL scaffolds supported the proliferation and chondrogenic differentiation of MSCs *in vitro* and when cell-seeded PVA/PCL scaffolds were implanted the animals treated showed improved healing of defects compared with untreated control and those which received cell-free scaffolds [85]. Combination of hydrogels and NPs containing a specific grow factor represents a suitable niche for the differentiation of transplanted hMSCs. hMSCs grown into hydrogels with NPs bearing TGF-β3 produced cartilage-specific ECM proteins such as collagen type II and glycosaminoglycan. The expression of these chondrocyte-specific ECM proteins clearly showed that hMSCs chondrogenesis occurred when the SCs were mixed with growth factor. Transplanted hMSCs into nude mice and rabbits arthritis defects proliferated easily and reliably, and switched without difficulty into differentiated chondrocytes capable of regenerating the wound tissue and finally remodelling the wound sites in the animals. These results showed that combined (cell and protein) delivery system helped to increase transplanted stem cell differentiation, thereby stimulating regeneration cascade events both *in vitro* and *in vivo* [86]. Another *in vivo* study was conducted in a swine model, and showed the applicability of biodegradable PCL NF scaffold, in this case, seeded with allogeneic chondrocytes or xenogeneic hMSCs. Thus, NF scaffolds effectively deliver therapeutic cells to cartilage lesions and support chondrogenic activity during the cartilage regeneration *in vivo*. The xenogeneic hMSCs-based treatment proved to repair cartilage defects and restore biomechanical functions of cartilage, suggesting the potential of MSCs xeno- or allo-grafting for cartilage tissue engineering [87].

## 3. Cell Encapsulation: Use in Regenerative Medicine

**Figure 1:**
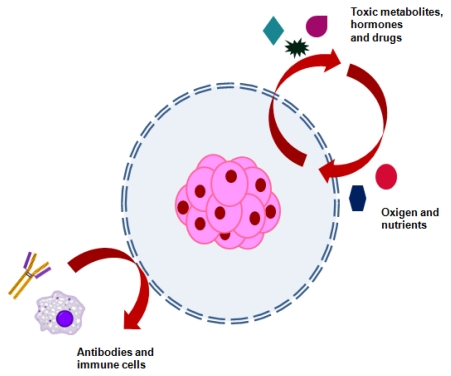
Schematic illustration of cell encapsulation technology. The semi-permeable membrane allows the bidirectional diffusion of nutrients, oxygen, therapeutic products and waste. At the same time this membrane avoids the entrance of immune cells and antibodies.

Cells in native tissue are embedded within a complex 3D microenvironment consisting of soluble molecules (cytokines and growth factors) and non-soluble factors (mainly ECM). The microenvironment not only provides structural integrity, but also controls numerous signal transduction processes that direct cell survival, cell cycle progression, and the expression of different phenotypes [88]. Cell encapsulation technology is based on the immobilization of cells within a semi-permeable membrane (Figure 1). This membrane protects the inner cells from both mechanical stress and the host immune system, while allowing the bidirectional diffusion of nutrients, oxygen and waste [89]. About 50 years ago, Chang proposed the protection of proteins and cells, by encapsulating them in a semi-permeable membrane [90]. Twenty years later, Lim and Sun presented the first implantable alginate-poly(l-lysine) microcapsules harbouring rat islet cells, that naturally secreted insulin, for the treatment of diabetes [91]. This was the first proof-of-principle study demonstrating the applicability of encapsulated cells.

The cellular recognition of biomaterials and the progression of subsequent cellular events are essentially based on integrin-mediated interactions, but depend in particular on the chemical and physical characteristics of biomaterials. The micro-fabricated biomaterials offer several advantages: (i) the individual parts of living cells exist in nano- or micro-scale lengths, and thus micro- or nano-fabrication techniques enable the recapitulation of their features; (ii) the micro-fabricated substrate can present simultaneously multiple adhesive or morphogenic signals in a small dimension simultaneously; and (iii) multiple parameters governing cell-biomaterial interactions could be analyzed separately with a small amount of analytes [92]. Microcapsule design must take into account a series of consideration including material biocompatibility, mechanical stability, permeability, size and durability. One main concern is the biocompatibility of the materials used, it should not interfere with cell homeostasis (encapsulated cells and surrounding host tissue) nor trigger an immune response in the host [93]. An interesting advance to predict biocompatibility was reported by de Vos *et al.*, where the measurement of the surface electrical charge, by means of zeta potential, was found to predict the interfacial reactions between the biomaterial and the surrounding tissue [94]. Mechanical stability must be maintained, because microcapsules withstand physical and osmotic stress and its breakage may lead to immune rejection of the encapsulated cells. Moreover, the capsule must have adjusted permeability in terms of entry and exit of the molecules. To gain a tight control of transport properties, it is essential that membrane wall thickness is uniform. The thinner the membrane, the faster is the nutrient diffusion and lower is the implant volume.

Another main concern is the immunoreactions against the capsule, which probability will trigger the immune system of the body. So encapsulated cells should be immunoprotected not only from immune cells but also from antibodies and cytokines [93]. An interesting approach using agarose capsules of different diameter developed by Sakai *et al.* revealed that cellular reaction to the smaller capsules was much lower than that to the larger capsules [95]. Thus, reduction in capsule diameter not only increased mechanical stability but also decrease the immune response against capsules. To fulfill the therapeutic goal of encapsulation, the biomaterial degradation rate should provide a sustained release of substances over a long period of time, into the surrounding environment, in a controlled fashion [93,96]. Other important issue is the technique used for encapsulation, because this process must be gentle and preserve cell integrity (*i.e.*, aqueous medium, no reactive species, no organic solvent). It is important to choose a reproducible method in which different parameter can be tightly controlled, for instance, permeability, size, and surface area. So far, different methods have been used to prepare cell-loaded microcapsules, including extrusion (electrostatic spray, air flow nozzle, and vibrating nozzle), emulsion/thermal gelation (agarose as core polymer), and microfluidic flow focusing approach [93,97,98].

**Figure 2:**
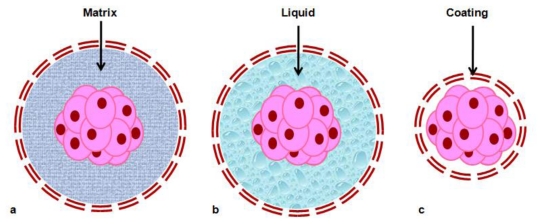
Models of cell encapsulation. (**a**) Matrix-core/shell microcapsules; (**b**) Liquid-core/shell microcapsules; (**c**) Cells-core/shell microcapsules.

The first assays of encapsulation were made using matrix-core/shell microcapsules. Cells were embedded in hydrogel matrix, often surface-modified by one or more external layers of different materials to improve permeability and stability properties, preventing cell release from the beads and ensuring biocompatibility (Figure 2). Despite that the hydrogels matrix allowed cell grown, the *in vivo* stability of electrostatic and hydrogen bonds maintaining the structure of microcapsules is limited, and the mechanical resistance of capsules is still a concern. Liquid-core/shell microcapsules where cells are embedded in liquid seem to allow better cell growth and protein production, because diffusion of gases and nutrients is higher in liquid than in gel, although their mechanical resistance is further diminished compared to matrix-core capsules. In this respect, semi-liquid cores and cells-core/shell where cells are directly surrounded by a coating layer appear to be good options. However, several questions pertaining to mechanical resistance (related to strength of the cell aggregates), the consequences of cell growth and death on the integrity of the immunoisolation barrier, and finally, long-term stability and performance, need to be addressed [93,97]. Figure 2 shows a schematic vision of the different models of encapsulations described here.

Alginate is the most common encapsulation material due to its intrinsic properties including biocompatibility, biosafety and permeability. The production of alginate cell microcapsules can be performed under safe and physiological conditions (e.g., physiological temperature and pH, use of isotonic solutions instead of cytotoxic solvents) and using good manufacturing practice (GMP) guidelines, a fact which potentiates the use of this technology in cell-based therapies [99–101]. Recently, Serra *et al.* evaluated benefits of microencapsulation in alginate when performing cell cultures. This work established that microencapsulation technology may be a powerful tool for integrating the expansion and cryopreservation of pluripotent hESCs. The 3D culture strategy developed herein represents a significant breakthrough towards the implementation of hESCs in clinical and industrial applications [99]. The potential of alginate microcapsules for transplantation is actually being tested, with two clinical trials currently under development using this material. One Phase I study is based on the encapsulation and transplantation of human islets in Type I diabetic patients (ClinicalTrials.gov Identifier: NCT00790257). And in the other, a Phase II clinical trial, the transplant is performed with encapsulated β cells for patients with Type I diabetes (ClinicalTrials.gov Identifier: NCT01379729). Table 1 shows the most frequently used materials in cell encapsulation for several diseases.

At the present, many studies focus on the encapsulation of SCs as a starting point for regenerative medicine, engineering tissue and gene therapy [98]. Therefore, the main objective of stem cell microencapsulation technology is to maintain the undifferentiated state of the cells and the controlled differentiation with the desired functions of those cells. Although the most employed material to develop the capsules is alginate, however, it has been reported that a novel microencapsulation technique fabricating self-assembled collagen—MSCs microspheres can be used as delivery devices for MSCs [128].

In summary, cell encapsulation allows continuous delivery of products from the cells for a longer period of time and, also, the transplantation of non-human cells, which could be considered as an alternative to the limited supply of autologous tissue. In addition, genetically modified cells can be immobilized to express any desired protein *in vivo* without the modification of the host genome [89,129] and achieve a transplant without immunosupression for the patients [97,130]. Nevertheless, successful application of microencapsulation requires interdisciplinary expertise ranging from biomedicine to materials science. Based on all the above, it has been proposed that layer by layer nanoscale coatings can be applied directly to the surface of the cell(s) to be encapsulated instead of using microencapsulation technology. These biocompatible nano-structured coatings serve in a similar fashion as the micro-capsules but have the advantage of easy diffusion of oxygen and essential nutrients. The encapsulation using nanotechnology also significantly reduces the volume of cells to be encapsulated [131,132].

## 4. Nanoparticle Systems for Tracking Transplanted SCs

The therapeutic potential of SCs in illnesses such as myocardial infarction, stroke, regeneration of bone and cartilage defects, spinal cord injury, graft-*versus*-host disease and blood disorders is widely recognized. In addition these cells are useful tools for modelling a broad range of diseases, both *in vivo* animal and *in vitro*. Among the intrinsic characteristic of SCs, their immunomodulatory properties and their low immunogenicity are essential features to be ideal candidates for cell therapy treatments. Adult MSCs have been shown to generate a local immunosuppressive microenvironment via cytokines activity and could produce key factors to inhibit both fibrotic and apoptotic phenomena [133].

Assuming that SCs may not trigger the immune response it is still important to consider how these cells can be guided to specific locations once they are transplanted into the patient. To monitor and evaluate the engraftment in the host, cells are labelled *ex vivo* to distinguish the implanted cells from the host tissue cells and follow their survival, migration, differentiation and regenerative impact in living subjects [134].

In these terms NP technology could help to track and localize transplanted cells. Nanocapsules are made with unique optical and/or magnetic properties to allow a non-invasive, accurate and real-time cell tracking [135]. Ideally, imaging technology used for SC tracking would have single-cell sensitivity allowing quantification of exact cell numbers at any anatomic location. *In vivo* imaging requires that a contrast agent associated with the transplanted cells exert an “effect size” sufficient for detection by the imaging hardware. Fluorescence labelling techniques are not sensitive enough to track *in vivo* injected cells because this approach requires invasive methods in order to address location of labelled cells [136]. For *in vitro* studies, microscopic optical imaging techniques include light and fluorescence microscopy, confocal microscopy, and two photon microscopy.

Conventional technologies and new approaches in the clinical radiology field have been used to track injected cells *in vivo.* X-ray, ultrasound and computed tomography (CT) have the disadvantage to require high concentrations of high density, high atomic number materials such as iodine, gadolinium, or metals, in order to achieve good contrast for image acquisition. Multimodality imaging techniques such as CT/positron emission tomography (PET), CT/single-photon emission computed tomography (SPECT), and magnetic resonance imaging (MRI)/PET combine different “anatomical” and “functional” methods. In this regard, MRI provides a very safe method for human *in vivo* imaging using a powerful magnetic field to align the nuclear magnetization of hydrogen atoms, which are responsible for the majority of MRI signals [137,138]. However, issues regarding to optimal concentration of image labelling agents and NP volume need to be studied, in order to improve the sensitivity and decrease background noise of the tracking methods.

### 4.1. Superparamagnetic Iron Oxide Particles (SPIO)

Combination of NPs and MRI are currently used for biomedical applications, however, contrasts agents used are mostly iron particles which accumulate in the liver. To improve imaging probes sensitivity and decrease particle toxicity, new strategies to coat the iron particles have been developed, consisting of nonstoichiometric microcrystalline magnetite cores, which are coated with dextrans (in ferumoxide) or siloxanes (in ferumoxsil). These SPIO are now used as MRI contrast probes for studying the fate of transplanted cells [139]. Commercially available dextran-coated SPIO (4.8–5.6 nm) ferumoxide NPs have been approved as intravenous contrast agents by the U.S. Food and Drug Administration (FDA) as Feridex^®^ and Endorem^®^ or as ultrasmall NPs (Combidex^®^, Sinerem^®^). Coating SPIO NPs with a protective layer of dextran or other polysaccharide helps to prevent their aggregation, induces the efficient internalization of the contrast agent into the cell and minimizes any deleterious effects on cellular function [140].

In summary, these novel contrast agents present the advantages of suitable magnetic saturation, superparamagnetic properties, and also their colloidal stability and biocompatibility [141,142]. In addition, they present less toxicity and do not adversely affect on cell physiology, differentiation, or cell migration.

#### 4.1.1. SPIO Integration into SCs

**Figure 3:**
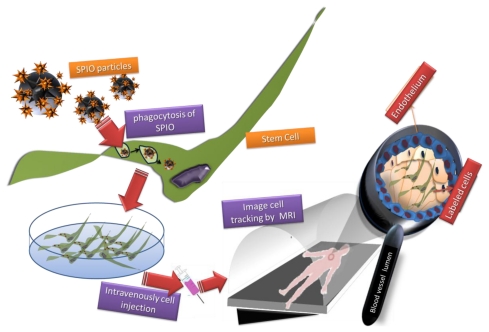
Scheme showing an overview of stem cell tracking: a work flow chart for labelling cells and introducing labelled cells into the human body include: (1) SPIO NPs are taken into the cell by an endocytosis process. This requires the coordinated action of some proteins like ENTH domain containing proteins, BAR superfamily proteins, ARF family small G proteins, proteins that nucleate actin polymerization, and dynamin superfamily proteins. The best-understood mechanism is clathrin-mediated endocytosis (CME). (2) Cells are cultured *in vitro* and *ex vivo*, and then, (3) injected intravenously into the human body. (4) SPIO-labelled SCs are then tracked in the body with MRI. SPIO NPs generate a signal that correlates with the cell location and permits non-invasive longitudinal tracking of cell therapies. In this example, MRI is used to track SPIO-labelled cells. In the case of Quantum dots, the same procedure is performed but using fluorescent tracking methods.

Iron oxide NPs are sequestered into SCs through endocytosis during *in vitro* cell cultivation, and accumulate in the endosomes. Depending on the size of them, iron oxide NPs can be internalized by several phenomena, including phagocytosis for SPIO and pinocytosis for ultrasmall superparamagnetic iron oxides particles (USPIO) and magnetodendrimers micron sized iron oxide particles (MPIOs) (Figure 3). This process is achieved in non-phagocytic cell types binding uptake-facilitating agents to the NP surface. Strategies to improve cell uptake include the use of transfection agents (e.g., poly-*L*-lysine or lipofectamine), electroporation [143], specific targeting and endocytosis of NPs through the use of transferrin receptors [144], magnetodendrimers [145], or transduction agents such as HIV-derived TAT protein which has been covalently bound to the particle surface to increase the transportation across the cell membrane [146–148]. According the cell line used and the NP employed it is important to assess specific parameters to achieve a highest uptake rate while not affecting the cell growth and function.

#### 4.1.2. SPIO: *In Vivo* Studies

Different studies using animal models have been carried out to evaluate the use of SPIO as a biomedical tool. SPIO labelling technique has been successfully used to study cell function and integration after transplantation in the cerebral nervous system (CNS), heart, liver, and kidney as well as in pancreatic islets of animal models.

Besides *in vivo* tracking conditions, SPIO NPs have been used to establish how intravenous delivery of human adipose stem cells (h-ADSC) can home to the radiofrequency ablated canine myocardial lesion and express a cardiomyocyte-like phenotype [149]. In diabetes mellitus allogeneic transplantation followed by MRI monitoring was used for the detection of SPIO-labelled pancreatic islets transplanted into the liver. Results showed graft rejection response over a 6-week period after transplantation [150]. In a same context, another study demonstrated the harmful effect of hyperglycemia in the survival-rates of islets grafts [151].

Cell therapies developed to cure neurological disorders such as multiple sclerosis (MS) have used SPI-labelled cells for high resolution monitoring of the bio-distribution of cells after transplantation into the CNS. Experimental autoimmune encephalomyelitis (EAE) mice were used to examine *in vivo* the functional response of glial-committed neural precursor cells (NPCs) labelled with SPIO. Results showed that labelled cells migrated over comparable distances and differentiated into glial lineages in the same way as unlabeled cells [152]. NPCs possess immune-modulating characteristics that are neuroprotective [153,154] and it is known that SPIO-labelling does not adversely affect NPC survival and differentiation within EAE brain [155,156]. Other *in vivo* studies have monitored the progression of neural SC therapy using NPs for up to 6 weeks [156,157]. A recent study showed that NPCs migrated when they were transplanted during either the acute or chronic disease phase and also observed the existence of differential NPC migration patterns; an important consideration for implementing future translational studies in MS patients with variable disease [158].

Although NPs have important applications for tracking SCs, however, there are potential scientific issues as well as safety issues that must be considered before clinical trials. In fact, clinical trials are hampered by basic research; nevertheless, few clinical trials using NPs are recruiting people at the moment.

### 4.2. Quantum Dots (QDs)

Quantum dots (QDs) are another class of nanomaterials with unique optical features, typically in the size range of 2–10 nm. They are inorganic fluorescent semiconductor NPs coated with an outer shell of a different material. First studies demonstrated the use of QDs as luminescent cell markers [159,160]. Currently, QDs are commercially available offering significant advantages over conventionally used fluorescent markers, due to their photostability, which allows long-term cell labelling and *in vivo* cell tracking. QDs are able to maintain fluorescent intensity in cell culture for a prolonged time, up to a few hours using confocal microscopy [161]. In fact, QDs are approximately 10–20 times brighter than fluorescent proteins [162]. Due to their long fluorescence lifetime QDs signal could be separated from background autofluorescence in cells or tissues. As well, the narrow emission and broad excitation spectrum of QDs allow quantification and simultaneous identification of multiple markers using single wavelength activation [159,160].

The major potential applications of QDs in regenerative medicine are their use for labelling and tracking of implanted SCs. Monitoring the cells *in vivo* after transplantation is essential for determining the efficacy of SC therapy. In a recent study, QDs were used to label ASCs, in combination with heparin, and analyzed their behaviour and organ-specific accumulation after having been transplanted in a mouse model of acute liver failure [163].

QDs could integrate into SCs through passive loading, receptor-mediated endocytosis or transfection. Rosen *et al.* showed that passive loading of QDs resulted in uniform diffused cytoplasmic labelling of a population of hMSCs and maintained similar proliferative and differentiation capacities compared to unloaded hMSCs. Then, passive loading of QDs in hMSCs is an effective method for uptake and did not significantly affect the features of the cells [164].

Progress in SC research has prioritized the refinement of cell-labelling techniques. Therefore, for effective labelling of MSCs and long observation by QDs *in vivo* it is necessary both to increase cellular uptake of QDs and to promote endosomal escape into the cytosol. In this matter, specific peptides such as cholera toxin [165], TAT-peptide [166], octa-arginine peptide phospholipids [167] or the polyamidoamine (PAMAM) dendrimer [168] have been used for efficient internalization of QDs. In addition, QDs have the capacity to be functionalized by means of cross-linking with proteins, streptavidin, antibodies, or other bioactive molecules [161,169], which allow QDs to be targeted to specific intracellular components and function as cell lineage tracing.

Different studies have been carried in order to analyze the effect of QDs on SCs properties; Slotkin *et al.* conducted an assay directing QDs labelling of mammalian stem and progenitor cells *in vivo* to study the developing mammalian central nervous system. Ultrasound guided *in vivo* delivery techniques were developed to specifically and efficiently label NSCs and progenitor cells (NSPCs) with QDs. QDs were found in the cell types generated by NSPCs at substantial distances away from the initial site of injection, suggesting that QD loading of NSPCs *in vivo* did not inhibit their migration or differentiation during the developmental cycle. Therefore, QDs labelling may be particularly useful for embryonic studies about the formation of nascent primordial layers [170].

QDs are also attractive nanomaterials for monitoring stem cell survival, location, and differentiation either *in vitro* or *in vivo* due to their inherent long-term fluorescence intensity [165–167]. Stem cells, by definition, are capable of self-replication and differentiation into multiple lineages; consequently many studies have been executed to show that these cells can be effectively labelled by QDs during both proliferation and multilineage differentiation for long term [171–173].

In conclusion, QDs based cell labelling can be used as a safe and effective means to facilitate *in vivo* trafficking of therapeutic cells. However, further studies are necessary to demonstrate the outcomes of QDs on long-term effects and their degradation products on SCs. In fact, the release of reactive oxygen species during the degradation of QDs contributes to its cytotoxicity [174,175]. Although, QDs are not completely innocuous it might be possible to coat the QDs in a way that circumvents their *in vivo* degradation.

## 5. Functionalized Peptide Nanostructures

After decades of fine-tuning many biological materials can now be designed and fabricated at the molecular level for particular uses. Because of their chemical complexity and structural sophistication, biological materials of natural origin have been more difficult to design and fabricate at the molecular level. However, over the last two decades the efforts of chemical scientists, chemical engineers, materials scientists and medical doctors have borne fruit and now a wide range of biomaterials for medical applications exist.

Peptide nanostructures containing bioactive signals that combine bioactivity for multiple targets with biocompatibility, improve the possibility to deliver proteins, nucleic acids, drugs and cells. Their chemical design versatility leads to a variety of possible secondary, tertiary and quaternary structures through folding and hydrogen bonding. In particular, β-sheet forming peptides have demonstrated the extraordinary ability to use intermolecular hydrogen bonding for the assembly of one-dimensional nanostructures. Furthermore, the design of self-assembly peptides for targeted functions can also include their modification with other biomolecular units such as sugars, lipid components and nucleic acid monomers. These oligopeptides, whilst designed for supramolecular self-assembly, could also serve to functionally mimic large proteins. For these reasons, self-assembling, nonimmunogenic peptides project as promising new therapeutics for human disease [176–178].

The first self-assembling peptide discovered was the EAK16, also named zuotin protein for its ability to bind to left-handed Z-DNA, which contains ubiquitous right-handed B-DNA and other DNA structures [179]. This peptide sequence (N-AEAEAKAKAEAEAKAK-C) has been extensively studied to create a class of simple β-sheet peptides [180]. These peptides are ionic self-complementary as a result of the presence of both positive and negative side chains on one side of the β-sheet and hydrophobic side chains on the other. These peptides have two distinctive sides, one hydrophobic and the other hydrophilic. The hydrophobic side forms a double sheet inside of a fibre and the hydrophilic side forms the outside of the nanofibres that interact with water molecules, forming an extremely high water content hydrogel, which can contain as high as 99.5% to 99.9% water (1–5 mg peptide per mL water, w/v). When dissolved in water in the presence of salt, they spontaneously assemble into well-ordered nanofibres and, then, further into scaffolds peptides, which form 3D nanofibre scaffolds that have been used in 3D cell tissue cultures.

Since EAK16 was discovered, many peptides and combinations are being studied. These peptide amphiphiles (PAs) not only have been used as scaffolds that provide structural support and bioactive signals, but these materials have also been moulded to allow for the study of the effects of matrix geometry on the behaviour of cells.

Some examples of different types of peptides that are being investigated and their possible application in regenerative medicine are:

1. The Ile-Lys-Val-Ala-Val (IKVAV) peptide sequence, derived from laminin, has been incorporated into PAs for applications in neural regeneration to enhance neural attachment, migration and neurite outgrowth. Neural progenitor cells cultured *in vitro* within networks of IKVAV PA quickly undergo selective and rapid differentiation into neurons with the formation of astrocytes being largely suppressed [26]. Control experiments using a mixture of soluble IKVAV peptide and PA nanofibres without the IKVAV epitope did not reveal this same response. These *in vitro* results suggested that the IKVAV PA may be a useful material in the treatment of spinal cord injury, where the formation of a glial scar, comprised primarily of astrocytes, prevents axonal regeneration after injury [181]. Mice treated with an injection of IKVAV PA solution 24 h after spinal cord injury showed that at the site of injection this solution formed nanofibres by self-assembly through electrolyte screening of the molecules. The material reduced cell death at the injury site and decreased the astrogliosis involving a hyperplasic state of astrocytes. The injected nanofibre gel also increased the number of oligodendroglia, the cells responsible for the formation of the myelin sheath around neurons in the central nervous system, at the injury site. Histological evidence was also obtained for the regeneration of descending motor axons as well as ascending sensory axons across the site of spinal cord injury in animals treated with the IKVAV PA [177]. This was accompanied by behavioural improvement in treated animals demonstrating enhanced hind limb functionality [182].
2. An interesting PAs with angiogenesis properties is the heparin-binding peptide amphiphile (HBPA), which was designed with a Cardin-Weintraub heparin-binding domain to specifically bind heparan sulphate-like gylcosaminoglycans (HSGAG). This glycosaminoglycan displayed charges on the HBPA molecules, triggering PA self-assembly into nanofibres that presented heparin on their surface. Moreover, they were able to capture many potent signalling proteins through their heparin-binding domains, including fibroblast growth factor 2 (FGF-2), bone morphogenetic protein 2 (BMP-2) and vascular endothelial growth factor (VEGF). This material was biodegraded and quickly remodeled into a well vascularised connective tissue without the addition of any exogenous growth factors [183–185].
3. Since nitric oxide has long been recognized as a possible solution to prevent complications of neointimal hyperplasia during angioplasty treatment in patients with atherosclerosis, PAs presenting heparin were mixed with diazeniumdiolate nitric oxide donors to prepare nitric oxide releasing nanofibre gels [186]. When applied to a rat carotid artery balloon injury model, the nitric oxide releasing PA nanofibre gels led to a reduction in neointimal hyperplasia by up to 77% compared with the controls, and also limited inflammation in the injury site [177].
4. PA nanofibres were explored as a means to functionalize the metal implants to enhance bioactivity and prompt tissue growth around the implant to assist in long-term implant fixation. A nickel-titanium (NiTi) alloy that is frequently used for stents, bone plates, and artificial joints was modified through covalent attachment of PA nanofibres using standard silanization and cross-linking chemistry [187,188]. Modifying the metal with RGDS-epitope presenting PAS leads to a significant increase in the number of adhered pre-osteoblastic cells cultured *in vitro*, whilst cells did not attach to the non-functionalized NiTi [187].
5. Branched RGDS-presenting PA nanofibres have been also used as scaffolds for ameloblast-like cells and primary enamel organ epithelial cells that initiate the process of enamel formation. When treated with branched RGDS PA nanofibres *in vitro*, these cells showed an enhancement in proliferation and increased their expression of amelogenin and ameloblastin, two proteins secreted by ameloblasts during enamel formation [189]. PAs have been also used in an *in vitro* scaffold for dental SCs, where SCs from human exfoliated deciduous teeth proliferate and secrete a soft collagen matrix when encapsulated within the PA, whilst dental pulp SCs differentiate into an osteoblast-like phenotype and deposit mineral when encapsulated within the gel [190].
6. The β-sheet peptide nanostructures have been also evaluated for the treatment of enamel decay, resulting in significant gains of net mineral within the lesions over the 5-day study. The peptide gels also nucleated the formation of *de novo* hydroxyapatite when incubated in mineralizing solutions [191]. The same peptides were evaluated as an injectable joint lubricant for the treatment of osteoarthritis [192].
7. Another peptide design that captures the self-assembling potential afforded by the β-sheet was prepared from monomers of alternating hydrophilic and hydrophobic residues, lysine and valine, respectively, flanking an intermittent tetrapeptide designed to mimic a Type II b-turn, termed a β-hairpin peptide. These peptides are designed to be hydrated in pure water, adopting a random coil conformation. Studies *in vitro* have found that these β-hairpin hydrogels can support survival, adhesion, and migration of fibroblasts, and can be used to encapsulate MSCs and hepatocytes. These gels have also been found to have inherent antimicrobial properties; showing selective toxicity to bacterial cells compared with mammalian cells [177,193,194].
8. The ionic self-complimentary peptides based on β-sheet-rich proteins from nature, prepared from sequences of alternating hydrophobic and hydrophilic residues, have the ability to support cell attachment to promote the survival, proliferation, differentiation and neurite growth for neural cells. Moreover, they were capable to promote differentiation of liver progenitor cells into hepatocyte spheroids and serve as scaffolds for human endothelial cells, as well as for chondrocytes and for osteogenic differentiation of hESCs [177,195–197].
9. Self-assembling peptides can also use conjugated aromatic groups such as carbobenzyloxy, naphthalene, or fluorenylmethyloxycarbonyl on the *N*-terminal end of di- and tri-peptides, demonstrating the formation of very stable, highly aunable hydrogels. A number of these sheets twist together to form nanotubes. These materials can also support chondrocyte survival and proliferation in both 2D and 3D [198,199].

Furthermore these systems can be used as templates for nanofabrication and biomineralization of synthetic replacements in biological tissues, with specific medical applications including diagnostic technologies. Indeed, functionalized peptide nanostructures are part of the immediate future with high relevance in regenerative medicine and drug delivery.

## 6. Nanoparticles for Gene and Drug Delivery into SCs

When a drug is introduced in the human body using traditional administration methods, a cascade of biotransformations occurs as result of its interaction with the biological environment. These processes are part of the drug metabolism. Drug metabolism is very complex and comprises oxidation, reduction, hydrolysis and conjugation reactions leading to final excretion from the body. Depending on the anatomical route, administered drugs are passing, upon absorption, through several tissues and organs (e.g., liver) before reaching the systemic circulation. In those organs, drugs may be subjected to chemical or enzymatic degradation. As a result, a higher dose of drug is necessary to ensure relevant therapeutic levels. Gene delivery methods using viral vectors, such as retroviruses, adenoviruses, and adeno-associated viruses, are extensively used and show superior transfection efficiency [200,201]. However, viral vectors have shown the disadvantages of potential immunogenicity and carcinogenicity, and have a complicated synthesis procedure. In this respect, the application of nanotechnology can be an answer toward the discovery and use of efficient and improved drug and gene delivery systems. NPs can be used as effective carriers of DNA, RNAi, proteins, peptides and small drugs for stem cell differentiation or survival [202].

In recent years, SC nanotechnology has emerged as a new exciting field since it overcomes some of the major limitations of conventional delivery system [203]. Novel nanomaterials, nanostructures, and nanotechnology have emerged to improve gene and drug delivery in SC-based therapies for injuries and degenerative diseases [204,205]. The plastic characteristic of nanomaterials implies that can be modified in size, morphology and solubility in order to increase cellular uptake. As a result, with the use of nanomaterials the pharmacokinetics of drugs can be controlled so that sustained therapeutic concentrations can be maintained at specific locations in the body with minimal side effects [206]. Non-viral gene vectors, including liposomes and cationic polymers, have received great attention because of their easy preparation, lack of immunogenicity, and ability to be modified for potential targeted delivery [207,208]. Among the cationic polymers, polyethylenimine [209] and polyethylene glycol [210] were reported to be effective transfection reagents due their easy synthesis and multiple modifications.

### 6.1. Nanoparticles for Drug Delivery into SCs

Drug delivery systems into stems cells are associated above all with the field of the cancer stem cells (CSCs). Carcinogenesis in humans is a multistage process, and the two major stages have been designated initiation and promotion. Although the biochemical basis for initiation and promotion remains to be established, recent research has provided important insights into potentially significant biologic mechanisms. Today is known that the initiation of carcinogenesis may result from cellular immortalization and the development of defects in the integrated control of stem cell proliferation and differentiation and that the promotion of carcinogenesis may result when such initiated SCs develop aberrant auto-regulatory growth-control properties. Understanding the role of CSCs during carcinogenesis, from tumour initiation to metastasis formation, has become a major focus in stem cell biology and in cancer research [211]. Nano-scale delivery systems provide bio-compatible platforms which combine the delivery of different therapeutic agents that act synergistically in one single vehicle. In addition, these systems can be multi-functionalized with targeting peptides or antibodies, which results in the selective targeting of the tumour site and CSCs. So, the drugs specifically reach the tumour tissues in the correct ratios and, thus, lower doses are expected to be required, which would reduce the number and severity of putative side effects. Eventually, multifunctional systems combined with CSC-targeting therapies should allow the specific and efficient delivery to tumour tissue, spare normal tissue and SCs, and result in the elimination of tumour cells, including stromal cells and CSCs.

In the past decade, NP-based drug delivery systems have shown exciting efficacy for cancer treatments due to their improved pharmacokinetics and biodistribution profiles via the enhanced permeability and retention (EPR) effect. Efficient delivery of therapeutics into tumour SCs to increase the intracellular drug concentration is a major challenge for cancer therapy due to drug resistance and inefficient cellular uptake. In this context, Du *et al.* designed a tailor-made dual pH-sensitive polymer-doxorubicin conjugate nanoparticulate system to overcome the challenges. The NP is capable of reversing its surface charge from negative to positive at tumour extracellular pH (±6.8) to facilitate cell internalization. Subsequently, the significantly increased acidity in subcellular compartments such as the endosome (±5.0) further promotes doxorubicin release from the endocytosed drug carriers. This dual pH-sensitive NP has shown enhanced cytotoxicity in drug-resistant cancer SCs, indicating its great potential for cancer therapy [212].

In addition, the combination of SCs and drug-loaded NPs for therapeutic applications in glioma therapy is a promising strategy, as NP could protect therapeutic agent and could allow its sustained release. The prognosis of patients with malignant glioma remains extremely poor, despite surgery and improvements in radio- and chemotherapies. However, new paradigms allowing tumour specific targeting and extensive intratumoral distribution must be developed to efficiently deliver NPs. Taking advantage into the fact that MSCs have a natural tropism toward brain tumours, Roger *et al.* proved that these cells could be used as NP delivery vehicles [213]. Two types of NPs loaded with coumarin-6 were investigated: (i) PLA-NPs and (ii) lipid nanocapsules (LNCs). Adult human marrow SCs efficiently internalized coumarin-6-PLA-NPs and coumarin-6-LNCs in a concentration and time-dependent manner. Moreover, cell viability and differentiation were not affected. Furthermore, these NP-loaded cells were able to migrate and distribute around the tumor mass by using the U87MG experimental human glioma in nude mice. These data suggest that MSCs can serve as cellular carriers for NPs in brain tumours and represent a promising tool as cell delivery systems in brain tumor therapy [213,214].

Low targeting efficiency is one of the biggest limitations for NPs drug delivery system-based cancer therapy. In another study MSCs were used as targeting vehicle and a silica nanorattle was employed as drug carrier [215,216]. The silica nanorattle-doxorubicin drug delivery system was efficiently anchored to the MSCs by specific antibody-antigen recognitions at the cytomembrane interface without any cell preconditioning. Up to 1500 NPs were uploaded to each MSC with high cell viability and tumour-tropic ability [217]. *In vivo* experiments proved that the burdened MSCs could track down the U251 glioma tumour cells more efficiently and deliver doxorubicin with wider distribution and longer retention lifetime in tumour tissues compared with free doxorubicin and silica nanorattle-encapsulated doxorubicin. The increased and prolonged doxorubicin intratumoral distribution further contributed to significantly enhanced tumour-cell apoptosis. This strategy has potential to be developed as a robust method for targeted tumour therapy with high efficiency and low systematic toxicity [217].

### 6.2. Nanoparticles for Gene Delivery into SCs

Tissue engineering can generally be divided into three main methodological areas of interest: direct gene delivery, cell therapy without involving genetic modification, and genetically modified cell-mediated therapy. The direct approach, gene delivery, focuses on an *in vivo* therapeutic transfer of genes to the host’s cells. These genes affect the host tissue *in situ* and induce tissue formation [218] and regeneration [219]. Cell therapy focuses on the use of naive cells mostly obtained from stem cell populations, which are placed in the site of injury with the goal of enhancing tissue regeneration [220,221]. Genetically engineered cell therapy employs both techniques: using tissue-specific or therapeutic genes and primary cells that overexpress these genes, it is possible to produce therapeutic proteins at sites of regeneration or to differentiate new cells into the desired cellular lineage and thus promote tissue regeneration [222]. Gene-modified MSCs possess superior characteristics of specific tissue differentiation [223], resistance to apoptosis [224], and directional migration [225]. Thus, nanotechnology is a useful tool for both generation and differentiation of SCs.

#### 6.2.1. Nanoparticle for Generation of Induced Pluripotent SCs

Reprogramming human somatic cells to induce pluripotent stem cells (iPSCs) by defined transcription factors (Oct3/4, Sox2, Klf4 and c-Myc) [226] has revolutionized the stem cell research community. iPSCs, like ESCs, have the potentiality to differentiate into any type of cell in the body but could be derived from patients’ own cells and therefore, transplantation may not require immunosuppressive therapy [227]. In addition, iPSCs research obviates the political and ethical dilemma associated with embryo destruction and ESC research. This remarkable discovery of cellular plasticity has important medical implications. iPSCs have great potential in human regenerative medicine and seem to be a good option in the treatment of several diseases, such as Parkinson's, macular degeneration or Type I diabetes, that require a homogenous population of mature and terminally differentiated cells.

**Figure 4:**
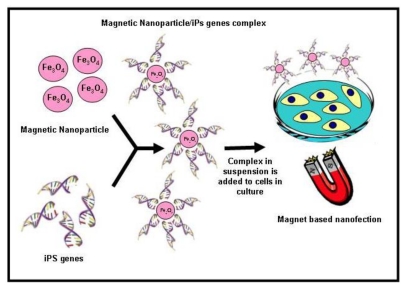
Generation of iPSCs from mouse embryonic fibroblasts by magnet-based nanofection. Plasmid DNAs containing reprogramming factors (Oct3/4, Sox2, Klf4 and c-Myc) were mixed with magnetic NP and the complexes were added to dishes that were applied to a magnetic field.

Although iPSCs offer great possibilities, there is a crescent concern regarding the procedures used to induce the pluripotentiality. The main techniques imply the use of viral vectors, such as retrovirus and lentivirus, which integrate the reprogramming factors into the host genomes and may increase the risk of tumour formation. Several non-integration methods have been reported to overcome the safety concern associated with the generation of iPSCs, such as transient expression of the reprogramming factors using adenovirus vectors or plasmids, and direct delivery of reprogramming proteins. Although these transient expression methods could avoid genomic alteration of iPSCs, they are inefficient [228]. The application of nanotechnology in iPSCs generation has attractive technological prospects; however, so far, few reports are closely associated with the use of nanotechnology to enhance the derivation of human iPSCs and labelling iPSCs cells for long-term tracking of their *in vivo* distribution. In this context, recently Lee and collaborators [229] generated iPSCs from fibroblasts using a non-viral magnetic NP-based transfection method that employs biodegradable cationic polymer PEI-coated super paramagnetic NP. These findings support the possible use of magnet-based nanofection for transient expression of transcription factors in somatic cells for efficient generation of iPSCs (Figure 4). After transfection, nanofection-mediated iPSCs showed ESC-like characteristics, including expression of endogenous pluripotency genes, differentiation of three germ layer lineages, and formation of teratomas. These results demonstrate that magnet-based nanofection may provide a safe method for use in generation of virus-free and exogenous DNA-free iPSCs, which will be crucial for future clinical applications in the field of regenerative medicine. Furthermore, Ruan *et al.* [230] successfully used fluorescent magnetic NPs (FMNPs) to label iPSCs for long-term observation and tracking.

#### 6.2.2. Nanoparticle as a Delivery System for SCs Differentiation

The ability to deliver biomolecules via an intracellular route, including growth factors, genes, proteins, and small chemicals, presents an excellent tool to direct SCs differentiation into specific cell types as bone, cartilage, muscle, adipose and cardiac. Some of these biomolecules/chemicals (i) have poor solubility, (ii) can be quickly cleaved by cellular enzymes, and (iii) have side effects when administered systemically. Biodegradable and biocompatible NPs able to target SCs and release the payload in their cytoplasm, with consequent activation of signalling cascades, will be of great interest.

**Figure 5:**
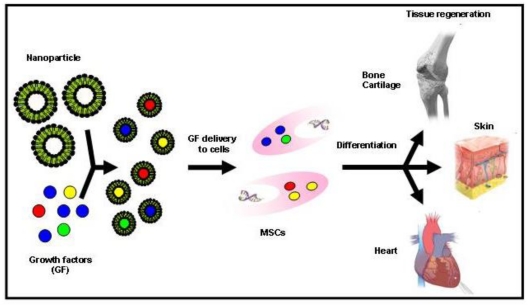
Growth factor-release based on a biomaterial approach to deliver signals to cells towards their differentiation for applications in tissue regeneration.

Growth factors are naturally occurring proteins capable of stimulating cellular proliferation, migration and/or differentiation into a specialized phenotype. Because they are involved in the regulation of several cellular functions, they can enhance the healing and regeneration processes of diverse tissues [231]. Nowadays, numerous growth factors are being identified, some of which produced by recombinant technology [232]. Due to the limited half-lives of many of these proteins *in vivo*, they are difficult to administer to sites of damaged tissue at therapeutic concentrations and for sustained periods of time. Thus, the way these molecules should be delivered to the injury site plays a crucial role for their success as therapeutic agents. To overcome the limitations of traditional methods of administration, several technologies using biomaterials have been explored to achieve a better control over the growth factor release. In this context, recently was reported a new approach for the delivery of vascular growth factors into hESCs, by incorporating growth factor-release particles in human embryoid bodies (EBs) [202]. The incorporation of these polymeric biodegradable particles had a minimal effect on cell viability and proliferation but a large impact on differentiation. The effect on vascular differentiation of particles containing growth factors was superior to the one observed by exposing EBs to large extrinsic doses of the same growth factors. These NPs could serve as a platform to deliver growth factors and other biomolecules within SCs (Figure 5).

Moreover, use of NPs, as delivery system, could be the best choice for bone regeneration, as there is no need for long-term overexpression of bone morphogenetic proteins (BMP) to induce valid bone formation. Transient overexpression of protein for a few weeks is sufficient to initiate the regeneration process. Indeed, many studies have shown the potential for MSCs that transiently overexpress BMP to induce bone formation on nanofibres [233] and chitosan-coated BMP-2 NPs [234]. Furthermore, several types of NPs were used as a delivery system to induce cartilage regeneration. Thus, for the safe and stable delivery of genes and to induce chondrogenesis, biodegradable PLGA NPs were used to mediate SOX9 gene delivery in hMSCs [235]. In addition, Park *et al.* [236] employed polycationic polymer, PEI polyplexed with a combination of SOX5, 6, and 9 fused to fluorescent protein coated onto PLGA NPs. SOX trio complexed with PEI-modified PLGA NPs led to a dramatic increase of hMSCs chondrogenesis *in vitro* culture systems. Recently, in the field of adipogenesis a systematic study in rat mesenchymal stem cells (RMSC) demonstrated that the biocompatible silica NP-insulin (SiNP) conjugates induce *in vitro* adipogenic differentiation [237]. Moreover, the biological activity of insulin conjugated to the SiNPs was not affected and the SiNPs could be used as biocompatible carriers of insulin for RMSC adipogenic differentiation, which would help to expand the new potential application of SiNPs in stem cell research. In addition, Yang *et al.* [238] achieved an efficient adipogenic differentiation of hMSC, *in vitro* as well of *in vivo* transplanted cells, using the adipogenic transcription factors C/EBP-α and C/EBP-β complexed with PEI coupled with biodegradable PLGA nanospheres. The expression of specific adipogenic genes and proteins in hMSCs was significantly elevated compared to the controls.

## 7. Toxicity Issues, Advantages and Limitations

The use of NPs in biomedical research implies a detailed consideration of their toxicity [239]. Thus, a full characterization of size, shape, charge, surface chemistry and material properties must be performed as it influences the production of free radicals and subsequent oxidative stress, inducing toxicity [240]. Nanomaterials may agglomerate *in vitro* or *in vivo* and may chemically degrade, making it difficult to relate systematically nanoparticle toxicity to such a diverse set of materials [241]. Several authors point out that *in vitro* and *in vivo* mechanisms of toxicity depend on the type of NPs [240,242,243]. For instance, toxicity of magnetic NPs might be due to several factors such as concentration, hydrodynamic size, surface charge, type of coatings, administration route or generation of iron-catalyzed reactive oxygen species. Evaluation of SPIO cytotoxicity in hMSCs [244,245], mESCs [246], and NSCs [247] showed in most cases that the internalization of these NPs by SCs did not affect cell viability, growth, or differentiation. Well characterized SPIO-labelled SCs showed a promising effect when combined with MRI; however, studies also demonstrate that this particles can in fact be transferred from SCs to non-SCs, such as macrophages like kuppfer cells in the liver [248]. In conclusion, more studies have to be performed to determine the cytotoxicity of NPs using appropriately validated analytical methods and well designed experimentation, so that nanomaterials can safely be used as therapeutics and diagnostic tools.

## 8. Conclusions

Significant advances achieved in both regenerative medicine and nanomedicine offer the promise of tissue and organ specific regenerative treatments. The development of functionalized bioactive scaffolds and NPs that promote cell proliferation, migration and differentiation has opened expectations for clinical application. However, some questions about safety and effectiveness of SC-based therapy and nanomaterials need to be addressed for widespread diagnostic and therapeutic use. A deep understanding of biological mechanisms responsible of regenerative processes based on nanomaterials-SC cell therapy is needed to obtain valuable information concerning cell behaviour, efficacy and undesirable effects of nanomaterials. Functionalization at molecular level of bioactive and biodegradable nanoscale filaments that mimic ECM must be explored in animal models before translation to clinic. After that, we believe clinical trials with current and novel nanomaterials will revolutionize application of regenerative medicine in patients.

## Figures and Tables

**Table 1:** Compilation of different materials used in encapsulation according to the disease for which they are intended. PEG (poly ethylene glycol); PLO (poly *L*-ornithine); PLL (poly *L*-lysine); PVA (poly vinyl alcohol); PLGA (poly *L*-lactide-co-glycolide).

DISEASES	MEMBRANE MATERIAL	Ref.
DIABETES	Alginate	[102]
	Alginate-PEG	[103]
	Alginate-PLO	[104,105]
	Alginate-PLL	[104]
	Alginate-Chitosan	[105,106]
	PVA	[107]
	Agarose	[108]
LIVER FAILURE	Alginate-Chitosan	[109]
	PEG	[110]
	PLL	[111]

CARDIOVASCULAR DISEASE	Alginate	[112]
	Fibrin	[113]
	Alginate-PLL	[96]
	Alginate-Chitosan	[114]

CNS DISEASE	Alginate-PLL	[115]
	PVA	[116]
	PLL-PLGA	[117]

BONE TISSUE ENGINEERING	Collagen	[118]
	Alginate-Chitosan	[119]
	Agarose-PEG	[120]
	Carboxymethyl xanthan	[121]
	Chondroitin sulfate-Agarose-PEG	[122]

CANCER	Alginate-PLL	[123]
	Hyaluronic acid	[124]
	Agarose	[125]
	Theracyte	[126]
	APA	[127]
